# Staged Reduction and Combined Anterior and Posterior Minimally Invasive Fixation for a Highly Unstable Open-Book Pelvic Ring Fracture: A Case Report

**DOI:** 10.7759/cureus.116895

**Published:** 2026-09-25

**Authors:** Tomohiro Miyashita, Atsushi Yunde

**Affiliations:** 1 Spine Center, Matsudo City General Hospital, Matsudo, JPN

**Keywords:** anterior subcutaneous internal fixation, external fixation, iliac screw, infix, sacroiliac ligaments

## Abstract

Vertically and rotationally unstable pelvic fractures are challenging to treat because they require accurate reduction and stable fixation, while conventional open procedures are associated with substantial morbidity. We report a highly unstable open-book pelvic ring fracture with marked displacement that was successfully treated using a staged reduction strategy and combined anterior and posterior minimally invasive fixation. A 27-year-old man presented with an open-book pelvic ring fracture. Computed tomography confirmed pubic symphysis reduction after external fixation; however, widening of the posterior aspect of the left-sided sacral fracture had become apparent, suggesting complete rupture of the posterior sacroiliac ligament. The sacral fracture was reduced and provisionally fixed while preserving rotational mobility of the left ilium. Pubic symphysis diastasis was reduced using anterior subcutaneous internal fixation in the supine position, followed by definitive posterior fixation in the prone position. A staged reduction strategy and combined anterior and posterior minimally invasive fixation achieved satisfactory reduction and favorable functional recovery, which was maintained at three years postoperatively.

## Introduction

Vertically and rotationally unstable pelvic fractures, classified as type 61C according to the Arbeitsgemeinschaft für Osteosynthesefragen (AO) Foundation/Orthopaedic Trauma Association (OTA) classification, are particularly challenging to treat because they frequently present with marked displacement and require accurate reduction and rigid stabilization. Although open reduction and internal fixation can achieve satisfactory reduction, they are associated with extensive blood loss, prolonged operative time, and a high risk of infection or wound complications [1,2]. In recent years, minimally invasive fixation techniques, including anterior subcutaneous internal fixation (INFIX) [3], have been introduced to reduce such complications while maintaining sufficient biomechanical stability [1,4-6]. However, in cases with marked displacement, achieving accurate reduction remains a major challenge. Here, we present a case of a highly unstable pelvic ring fracture with marked displacement, in which a staged reduction strategy and combined anterior and posterior minimally invasive fixation achieved satisfactory reduction and favorable functional recovery.

## Case presentation

A 27-year-old man was injured when he fell from a height of 1 m while operating a reach forklift truck in reverse. On arrival at our hospital, he was in hemorrhagic shock, with an initial blood pressure of 89/72 mmHg, heart rate of 149 beats/minute, hemoglobin level of 9.3 g/dL, and lactate level of 5.7 mmol/L. Initial radiographs and computed tomography (CT) revealed an open-book pelvic ring fracture with a nondisplaced fracture of the right sacral ala (AO/OTA type 61C2.3, Young-Burgess anteroposterior compression type 3 (APC3)) together with a Morel-Lavallée lesion [7] in the lumbar region, fractures of the left femur and left humerus, and a left shoulder dislocation (Figure 1). The Morel-Lavallée lesion was managed conservatively.

**Figure 1:**
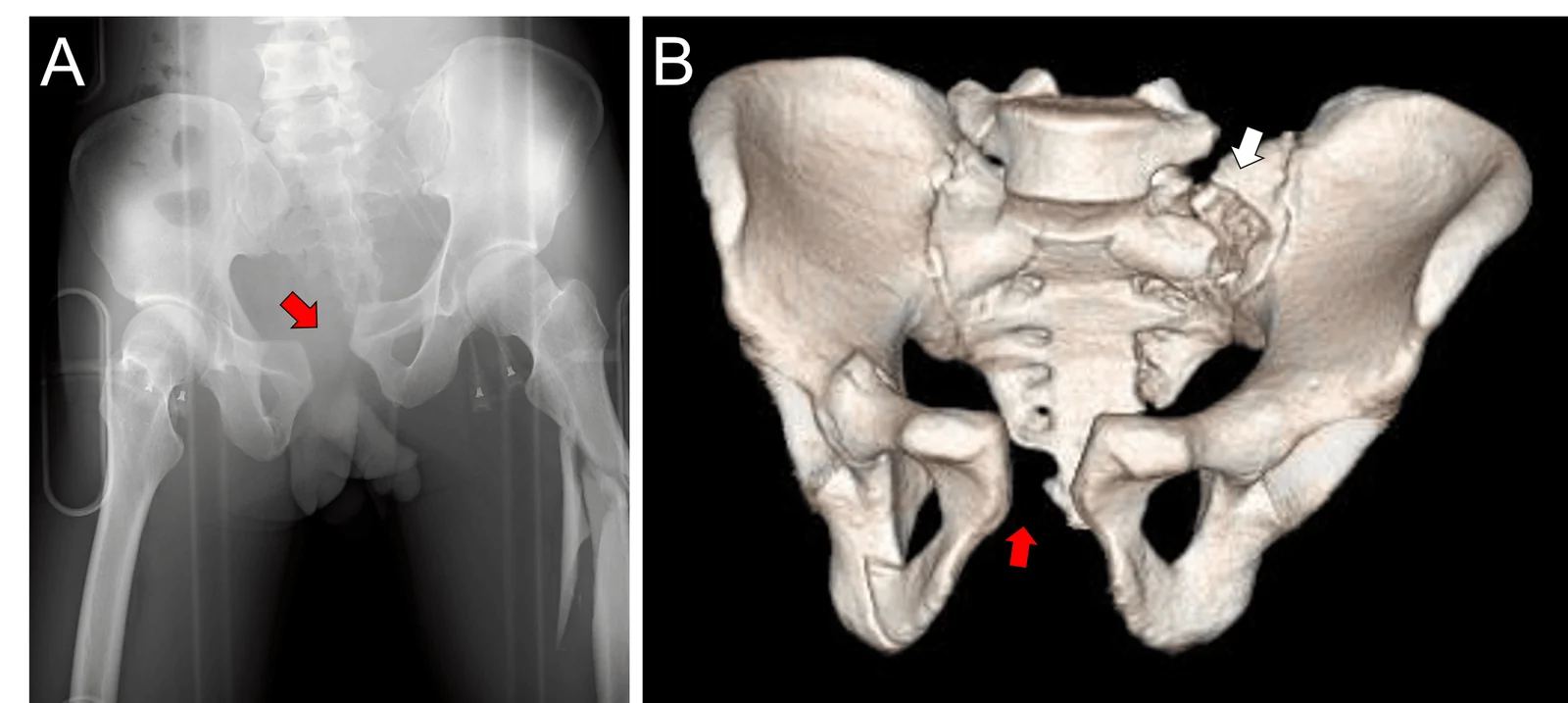
Initial radiograph and computed tomography image. (A) Anteroposterior radiograph and (B) three-dimensional reconstructed computed tomography image showing pubic symphysis diastasis (red arrows) with widening of the anterior aspect of the left-sided sacral fracture (white arrow) and a nondisplaced fracture of the right sacral ala.

The patient became hemodynamically stable after preperitoneal packing, transcatheter embolization of the internal iliac artery, and blood transfusion. Immediately after the embolization, the orthopedic team reduced the pubic symphysis diastasis under fluoroscopic guidance and stabilized the pelvis using an iliac crest external fixator. Post-external-fixation CT confirmed satisfactory reduction of the pubic symphysis but revealed widening of the posterior aspect of the left-sided sacral fracture that had become apparent following anterior reduction, suggesting complete rupture of the posterior sacroiliac ligament (Figure 2).

**Figure 2:**
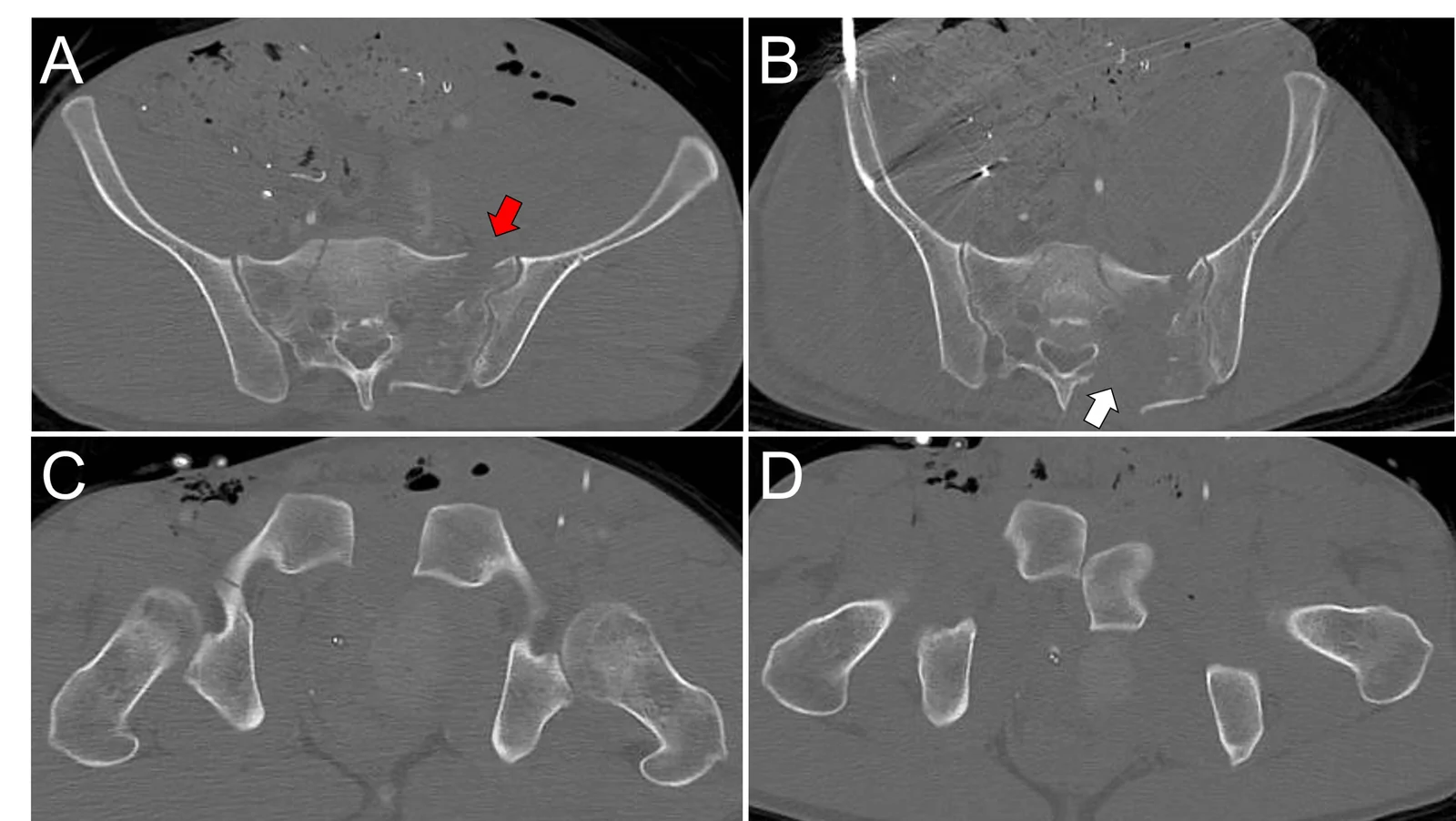
Computed tomography images before and after external fixation. Axial computed tomography images showing (A) widening of the anterior aspect of the left-sided sacral fracture (red arrow) before external fixation and (B) widening of the posterior aspect of the fracture (white arrow) after external fixation, as well as (C) pubic symphysis diastasis before external fixation and (D) its reduction after external fixation.

Combined anterior and posterior reduction and fixation of the pelvic fracture was performed 15 days after the injury, as the patient required intensive care and surgery for the associated fractures during this period. First, after a posterior midline incision and bilateral subcutaneous tissue exposure, a single iliac screw from the Ballast™ Spinal System (Medtronic, Minneapolis, MN, USA) was inserted into each ilium through the fascia in the prone position, with a closed-head screw used for the left ilium. The iliac screws were connected with a 5.5-mm rod through offset connectors, with the set screw of the left closed-head iliac screw intentionally left untightened (Figures 3A, 3B). Under fluoroscopic guidance, we reduced the widening of the left-sided sacral fracture by applying compression with a compressor hooked onto the left offset connector and a rod gripper, using the disappearance of fracture widening as the fluoroscopic criterion for reduction (Figure 3C). The pelvis was then provisionally fixed by tightening the set screw of the left offset connector while preserving rotational mobility of the left ilium.

**Figure 3:**
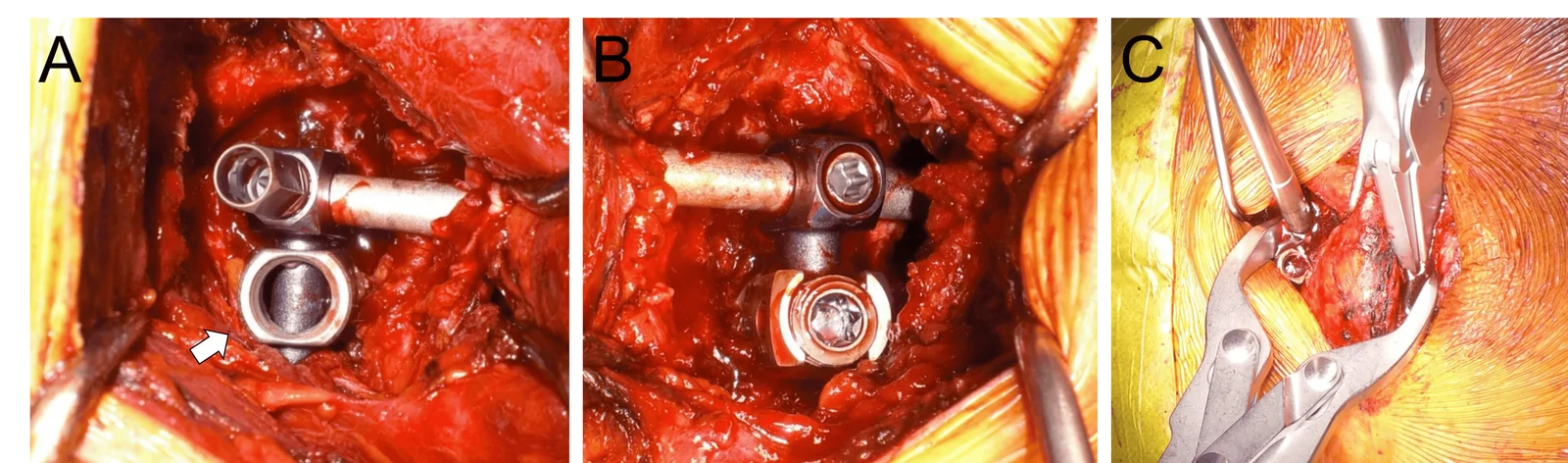
Intraoperative images. Intraoperative images showing iliac screws connected with a rod through offset connectors, (A) with the set screw of the left closed-head iliac screw intentionally left untightened (white arrow) and (B) with the set screw of the right iliac screw tightened, and (C) compression of the left-sided sacral fracture using a compressor hooked onto the left offset connector and a rod gripper.

After provisional closure of the posterior incision, the patient was repositioned to the supine position. The pubic symphysis diastasis was reduced under fluoroscopy and fixed using the INFIX technique [3] with the Solera™ Voyager™ 5.5/6.0 Spinal System (Medtronic, Minneapolis, MN, USA). The INFIX technique used 8.5-mm screws, with the screw heads and 5.5-mm rod positioned above the fascia. The rod was cut to an appropriate length to avoid excessive lateral protrusion beyond the screw heads, which could injure the lateral femoral cutaneous nerves. The patient was then repositioned to the prone position. The offset connectors and rod were removed, and additional iliac screws were inserted cranial to the previously placed screws. Finally, the cranial and caudal pairs of iliac screws were connected with separate 5.5-mm rods (Figure 4A). No additional compression was applied before final tightening. The total operative time, including the time required for repositioning, was 529 minutes, and the estimated blood loss was 161 g. Postoperative CT showed satisfactory reduction of the left-sided sacral fracture and pubic symphysis diastasis (Figures 4B, 4C). The pelvic asymmetry value decreased from 46.9 mm preoperatively to 21.0 mm postoperatively, defined as the difference in length between two diagonal lines drawn from the inferior margins of the sacroiliac joint to the contralateral inferior margins of the teardrop (Figures 1A, 4A) [8-10].

**Figure 4:**
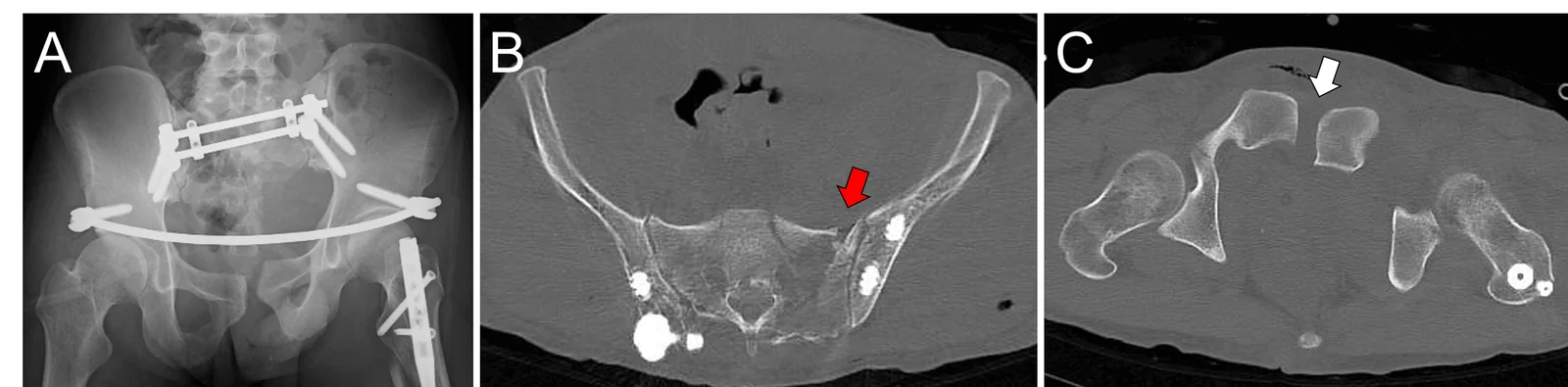
Postoperative images. (A) Anteroposterior radiograph demonstrating satisfactory reduction of the pelvic fracture. Axial computed tomography images showing satisfactory reduction of (B) the left-sided sacral fracture (red arrow) and (C) the pubic symphysis with slight residual widening (white arrow).

The patient was allowed to sit on the bed on the first postoperative day. Ambulation with non-weight-bearing on the left leg was not possible because the left humeral fracture and shoulder dislocation prevented crutch use. No postoperative complications, including infection, were observed. Full weight-bearing ambulation was permitted eight weeks postoperatively. All instrumentation, including the anterior INFIX, was removed after confirmation of bone union on CT one year postoperatively (Figure 5).

**Figure 5:**
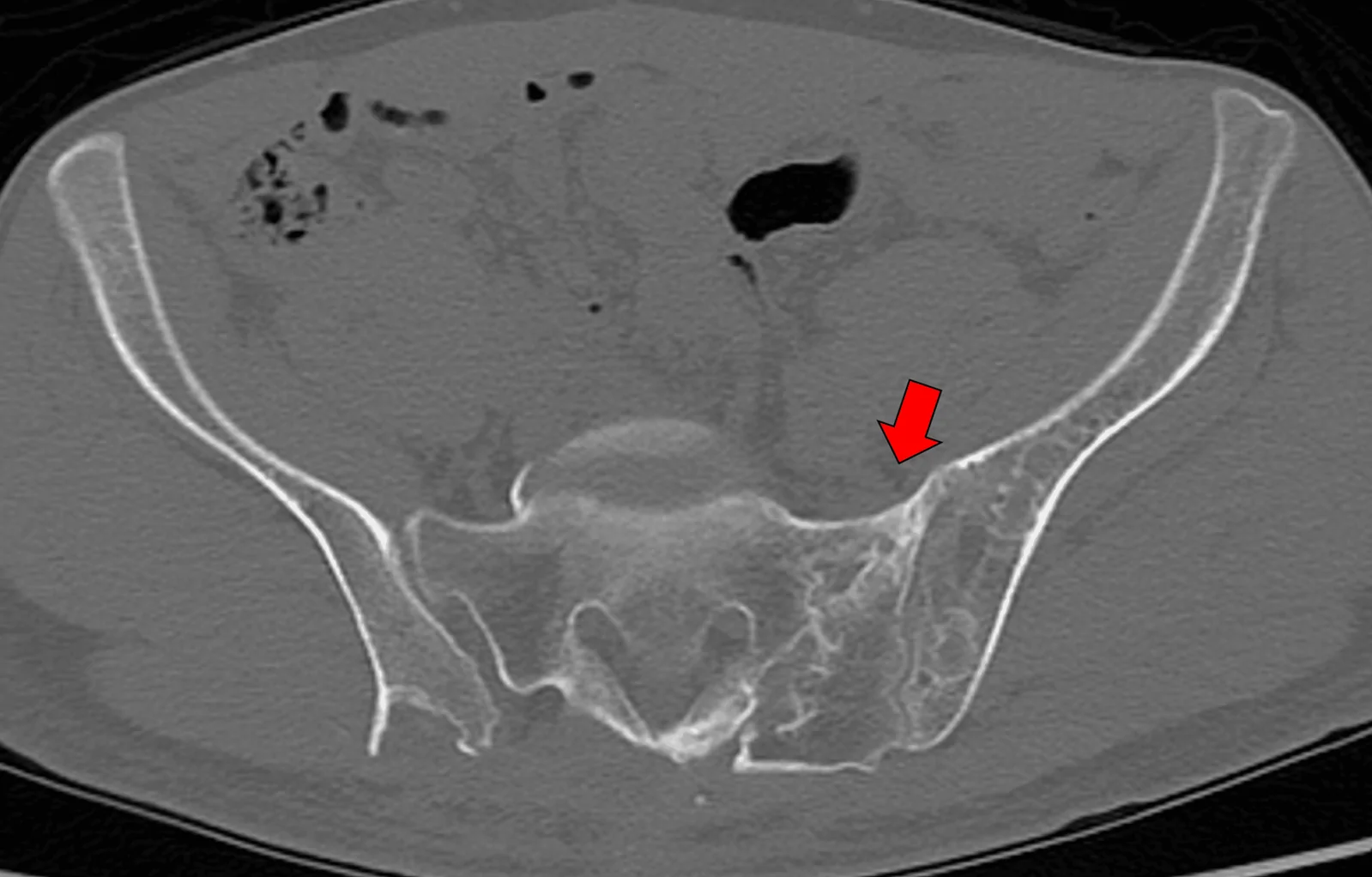
One-year postoperative computed tomography image. Computed tomography image obtained after implant removal at one year postoperatively, demonstrating bone union of the left-sided sacral fracture (red arrow).

Three months after implant removal, his Majeed score was 87 out of 100, indicating an excellent clinical grade [11]. He was able to ambulate stably with only slight numbness of the left thigh and had no limitations in activities of daily living at three years postoperatively. This symptom had not been reported before or after surgery or before or after implant removal.

## Discussion

This case involved a highly unstable open-book pelvic ring fracture (AO/OTA type 61C2.3, Young-Burgess APC3) with marked displacement, which was successfully treated with a staged reduction and combined anterior and posterior minimally invasive fixation. By using stepwise reduction techniques and positional changes during surgery, satisfactory reduction and fixation were achieved with minimal invasiveness. This report highlights the feasibility and utility of this approach in managing complex pelvic fractures.

Reduction strategy and its implications

AO/OTA 61C and Young-Burgess APC3 fractures are characterized by both rotational and vertical instability. In managing such unstable injuries, accurate reduction is critical for functional outcomes. Therefore, meticulous preoperative planning and intraoperative strategies are essential. In this case, although definitive fixation was planned as a second-stage procedure after external fixation, post-external-fixation CT revealed widening of the left-sided sacral fracture, suggesting complete rupture of both the anterior and posterior sacroiliac ligaments and prompting modification of the reduction and fixation strategy. A stepwise approach was indicated: posterior reduction and provisional fixation preserving rotational mobility of the left ilium were performed in the prone position. Anterior reduction and fixation (INFIX) [3] were then performed in the supine position, followed by repositioning the patient to the prone position for definitive posterior fixation. This sequence allowed reduction forces to be applied in a controlled, direction-specific manner.

Pelvic tilt and iliac displacement

In open-book pelvic fractures, in which the sacroiliac joint opens anteriorly, anterior pelvic tilt may create an appearance of vertical displacement of the iliac crest and vertical offset of the pubic symphysis on anteroposterior radiographs due to projection; however, this finding should be distinguished from true vertical shear injuries [12]. In such fractures with intact posterior sacroiliac ligaments, cranial displacement of the ilium is typically reduced with INFIX alone [13]. However, in the present case, which involved complete rupture of the posterior sacroiliac ligament, the displacement could not be reduced with INFIX alone, and even our technique could not fully counteract the effect of pelvic tilt. One conceivable solution was to push and tilt the ilium anteriorly using a set-screw driver for the left offset connector during the reduction of the left-sided sacral fracture diastasis.

Other reduction methods

It may be possible to reduce and fix such complex fractures using less invasive methods if screws with special functions that allow independent locking of the polyaxial head and rod are available. This design provides the flexibility of a polyaxial screw while enabling reduction control comparable to that of a monoaxial screw. In transiliac rod and screw fixation [6] modified using such screws, rotational reduction of anterior widening at the sacral fracture can be achieved by manipulating one iliac screw with an attached driver shaft as a joystick on the injured side. While maintaining this reduced position, the polyaxial head of the other ipsilateral iliac screw is then locked after rod insertion, thereby converting it into a monoaxial screw to maintain the reduction. Compression is subsequently applied to the monoaxial screw head to reduce the horizontal displacement.

Reduction using a Starr frame followed by fixation is another alternative [10,14,15]. The frame allows multiplanar reduction of the injured hemipelvis through manipulation of external fixator pins. Once the pelvis is reduced and stabilized on the frame, definitive fixation can be performed in a controlled manner. However, this approach requires specialized equipment, multiple procedural steps, and advanced technical expertise.

Limitations

This study had several limitations. First, as a single case report, generalizability is limited. Prospective studies with long-term follow-ups are needed to establish standardized treatment strategies. Second, the present method required multiple patient repositionings and was time-consuming. The use of specialized instruments and the incorporation of three-dimensional preoperative planning and intraoperative navigation may lead to simpler and less invasive approaches.

## Conclusions

A staged reduction strategy and combined anterior and posterior minimally invasive fixation provided a practical approach for managing this highly unstable open-book pelvic fracture with marked displacement. The sequential use of posterior provisional fixation, anterior fixation, and definitive posterior fixation allowed controlled reduction while preserving rotational mobility of the injured ilium during the procedure. This approach may be a useful option when satisfactory reduction is difficult to achieve with a single reduction maneuver or fixation method.
